# TLR2 and Dectin-1 Signaling in Mouse Hematopoietic Stem and Progenitor Cells Impacts the Ability of the Antigen Presenting Cells They Produce to Activate CD4 T Cells

**DOI:** 10.3390/cells9051317

**Published:** 2020-05-25

**Authors:** Alba Martínez, Cristina Bono, Daniel Gozalbo, Helen S. Goodridge, M. Luisa Gil, Alberto Yáñez

**Affiliations:** 1 Departamento de Microbiología y Ecología, and Estructura de Recerca Interdisciplinar en Biotecnologia i Biomedicina (ERI BIOTECMED), Universitat de València, 46100 Burjassot, Spain; alba.martinez@uv.es (A.M.); cristina.bono@uv.es (C.B.); daniel.gozalbo@uv.es (D.G.); m.luisa.gil@uv.es (M.L.G.); 2 Board of Governors Regenerative Medicine Institute, and Research Division of Immunology, Department of Biomedical Sciences, Cedars-Sinai Medical Center, Los Angeles, CA 90048, USA; helen.goodridge@csmc.edu

**Keywords:** hematopoietic stem and progenitor cells, TLR2, Dectin-1, antigen presenting cells, CD4 T cells, innate immune memory

## Abstract

Microbial recognition by pattern recognition receptors (PRRs) expressed on hematopoietic stem and progenitor cells (HSPCs) not only activates myelopoiesis but also programs the function of the monocytes and macrophages they produce. For instance, changes in HSPC programming modify the ability of macrophages derived from them to produce inflammatory cytokines. While HSPCs exposed to a TLR2 agonist give rise to tolerized macrophages (lower proinflammatory cytokine production), HSPCs treated with Dectin-1 ligands produce trained macrophages (higher proinflammatory cytokine production). However, nothing is known about the impact of HSPC exposure to microbes on the function of antigen presenting cells (APCs). In this study we evaluated whether treatment of murine bone marrow HSPCs with a TLR2 or Dectin-1 ligand impacts the antigen presenting capacity of APCs derived from them in vitro. Following activation with microbial ligands or *Candida albicans* yeasts, APCs derived from TLR2/Dectin-1-programed HSPCs exhibit altered expression of MHCII (signal 1), co-stimulatory molecules (CD40, CD80 and CD86; signal 2) and cytokines (TNF-α, IL-6, IL-12 p40 and IL-2; signal 3). Moreover, APCs derived from TLR2/Dectin-1-programed HSPCs prime enhanced Th1 and Th17 responses, which are important for antifungal defense, in CD4 T cell cocultures. Overall, these results demonstrate for the first time that microbial detection by bone marrow HSPCs can modulate the adaptive immune response by inducing the production of APCs with an altered phenotype.

## 1. Introduction

Hematopoietic stem and progenitor cells (HSPCs) are increasingly being appreciated to be important players in the fight against microbial infections, due to their ability to produce more innate immune cells to replace those consumed during the infection process and boost the number of responding cells. Besides sensing inflammatory mediators released by immune and stromal cells activated by pathogens, HSPCs can also directly detect microbial ligands. Several functional pattern recognition receptors (PRRs) are expressed on HSPCs, such as Toll-like receptors (TLRs) and Dectin-1, and engagement by their ligands induces HSPCs to proliferate and differentiate towards the myeloid lineage [1,2]. For instance, our group has demonstrated that interaction of *Candida albicans* with HSPCs induces their proliferation and differentiation into functional myeloid cells in a TLR2- and Dectin-1-dependent manner [3].

Remarkably, however, TLR2 and Dectin-1 signaling instruct very different functional programing in HSPCs. HSPCs treated in vitro with Pam_3_CSK_4_ (a TLR2 agonist) give rise to macrophages with a reduced ability to produce inflammatory cytokines (tolerized response) [4]. By contrast, HSPCs treated in vitro with β-glucans (a Dectin-1 agonist found in the cell wall of fungi) or whole *C. albicans* yeasts give rise to macrophages with an enhanced ability to produce inflammatory cytokines (trained response) [5]. Therefore, macrophages derived from HSPCs exposed to microbial ligands display changes in their functional phenotype. These data indicate that innate immune memory, which has been described in monocytes and results from long-lasting epigenetic and metabolic changes that alter their functional properties, also occurs in HSPCs, and thus, this phenomenon might contribute to the durability of innate immune memory [6]. Consistent with this, in vivo studies have demonstrated that β-glucans and the Bacillus Calmette-Guérin (BCG) vaccine impact progenitor programming and train monocyte and macrophage responses, and most importantly, have shown that trained HSPCs have the capacity to induce heterologous protection against secondary infections [7,8,9].

Myeloid cells are critical for successful immune responses against pathogens. In addition to directly controlling pathogens, they act as antigen presenting cells (APCs) that process pathogen antigens and present them on MHCII molecules to activate CD4 T cells to initiate adaptive immunity. T helper (Th) 1 and Th17 responses are particularly important to control fungal infections and some bacterial infections [10]. However, little is known about the effects of innate immune memory on the activation of the adaptive immune system. In this study, we evaluated whether in vitro treatment of murine bone marrow HSPCs with a TLR2 or Dectin-1 ligand impacts the function of the APCs derived from them. To this end, we evaluated how treatment of HSPCs with TLR2 and Dectin-1 ligands impacts the three signals that APCs derived from them deliver to activate CD4 T cells: MHCII (responsible for antigen presentation to CD4 T cells), costimulatory molecules, and cytokines. We also evaluated the ability of these APCs to induce CD4 T cell proliferation and Th1 and Th17 polarization upon presentation of: (i) ovalbumin (OVA) peptide in cocultures with OVA-specific CD4 T cells from OT-II transgenic mice, and (ii) antigens derived from *C. albicans* cells in cocultures with CD4 T cells from wild-type mice.

## 2. Materials and Methods

### 2.1. Mice

C57BL/6 mice were purchased from Envigo and The Jackson Laboratory. OVA peptide (323–339) specific TCR-transgenic mice (OT-II) were purchased from The Jackson Laboratory. Mice between 8 and 24 weeks old were used, and all the studies were carried out in strict accordance with regulations of the University of Valencia and Cedars-Sinai Medical Center Institutional Animal Care and Use Committees.

### 2.2. Microbial Components and Fungal Cell Preparation

The stimuli used were the TLR2 ligand Pam_3_CSK_4_, the Dectin-1 agonist depleted zymosan (a *Saccharomyces cerevisiae* cell wall preparation that has been treated with hot alkali to remove its TLR-stimulating properties), both from Invivogen (Toulouse, France), and inactivated *C. albicans* yeasts from PCA2 and ATCC 26555 strains prepared as follows. Starved fungal cells were inoculated (200 µg dry weight of cells/mL) in a minimal synthetic medium and incubated for 3 h at 28 °C with shaking to promote a yeast-form growth. For inactivation, yeast cells were resuspended (20 × 10^6^ cells/mL) in BD Cytofix™ Fixation Buffer (BD Bioscience, San Jose, CA, USA) containing 4% paraformaldehyde and incubated for 30 min at room temperature. After treatment, fungal cells were extensively washed in PBS, brought to the desired cell density and maintained at −80 °C in dry sediment until use.

### 2.3. Isolation of Hematopoietic Stem and Progenitor Cells and APC Differentiation

Mouse HSPCs were isolated as lineage-marker negative cells (Lin^−^ cells) from bone marrow. Briefly, murine bone marrow was obtained by flushing the femurs and tibias; cells were depleted of lineage positive cells by immunomagnetic cell sorting using MicroBeads (Miltenyi Biotec, Madrid, Spain): bone marrow cells were labelled with a cocktail of antibodies against a panel of lineage antigens [CD5, CD45R (B220), CD11b, Gr-1 (Ly-6G/C), 7-4, and Ter-119], and then, cells were purified by negative selection according to the manufacturer’s instructions. Depletion of lineage positive cells (T cells, B cells, monocytes/macrophages, granulocytes, and erythrocytes) from the bone marrow enriches for CD117^+^ HSPCs. Lin^−^ cells were cultured in 24-well plates at a density of 500,000 cells/well in complete cell culture media [RPMI 1640 supplemented with 10% heat-inactivated FBS and 1% penicillin-streptomycin-glutamine stock solution (Gibco, Barcelona, Spain)] containing 20 ng/mL rmGM-CSF (Preprotech, London, UK) and challenged with the indicated stimuli during the first 24 h. Lin^−^ cells were thoroughly washed with PBS, transferred to a 15 cm culture dish and continued culture in 20 mL of complete cell culture media containing 20 ng/mL rmGM-CSF for 6 days. The media was changed on day 3 as follows: the existing media was reduced to 5 mL and 15 mL fresh complete media + 20 ng/mL rmGM-CSF was added. Adherent cells were harvested on day 6, counted, plated in complete cell culture media containing 20 ng/mL rmGM-CSF, and rested for 24 h prior to stimulation.

### 2.4. APC and CD4 T Cell Co-Culture

APCs were plated in 48-well plates at a density of 80,000 cells/well. APCs were treated with OVA_323–339_ peptide (7 µg/mL) (Invivogen), the specific antigen for CD4 T cells from OT-II mice, for 30 min and then challenged with the APC maturation stimuli Pam_3_CSK_4_ (100 ng/mL) or depleted zymosan (100 µg/mL) (both from Invivogen) for 24 h. Alternatively, APCs were treated with *C. albicans* PCA2 or ATCC 26555 inactivated yeasts, which provide both antigens and APC maturation stimuli, at a ratio 1:12 (murine cell:yeast) for 24 h. CD4 T cells were isolated from spleens of OT-II mice (for OVA-loaded APC co-cultures) or C57BL/6 mice (for yeast-loaded APC co-cultures) by immunomagnetic negative selection (STEMCELL Technologies, Grenoble, France). Purified cells were labelled with CFSE (Invitrogen, Barcelona, Spain) and resuspended in a cell culture media supplemented with 1 M sodium pyruvate and 50 nM 2-ME. APCs were washed twice with PBS and 400,000 CD4 T cells/well were added 1:5 ratio (APC:T cell). Following 3 or 4 days of coculture, T cells were harvested for flow cytometry analysis and cytokine production measurements. 

### 2.5. Antibodies and Flow Cytometry

Flow cytometry analyses were performed on a BD Fortessa Analyzer (BD Biosciences) and the data was analyzed with FACSDiva and FlowJo 10 software. Antibodies used were purchased from BD Bioscience [CD11b (clone M1/70), CD11c (clone N418), CD4 (clone RM4-5), CD69 (clone H1.2F3)] or from Biolegend [CD40 (clone 3-/-23, isotype control rat IgG2a), CD80 (clone 16-10A1, isotype control hamster IgG), CD86 (clone GL1, isotype control rat IgG2a), MHCII (clone M5/114.15.2, isotype control rat IgG2b), CD44 (clone IM7)].

### 2.6. Cytokine Measurements

APCs were plated in 96-well plates at a density of 50,000 cells/well and challenged with the indicated stimuli for 24 h. Cell-free supernatants were then harvested and tested for TNF-α, IL-6, IL-12 p40, and IL-2 release using commercial ELISA kits (Biolegend, San Diego, CA, USA). Total T cells were harvested from APC cocultures, plated in 96-well plates and restimulated with 50 ng/mL PMA plus 500 ng/mL ionomycin for 24 h and cell-free supernatants were collected. IL-17A and IFN-γ production was quantified using commercial ELISA kits (Biolegend).

### 2.7. Statistics

Statistical differences were determined using two-tailed Student’s *t*-test. Data are expressed as mean ± SD. Significance was accepted at * *p* < 0.05, ** *p* < 0.01, and *** *p* < 0.001 levels.

## 3. Results

### 3.1. HSPC Exposure to Pam_3_CSK_4_ or Depleted Zymosan Alters MHCII Levels, Co-Stimulatory Molecule Expression, and Cytokine Release by the APCs Derived from Them In Vitro

**Figure 1:**
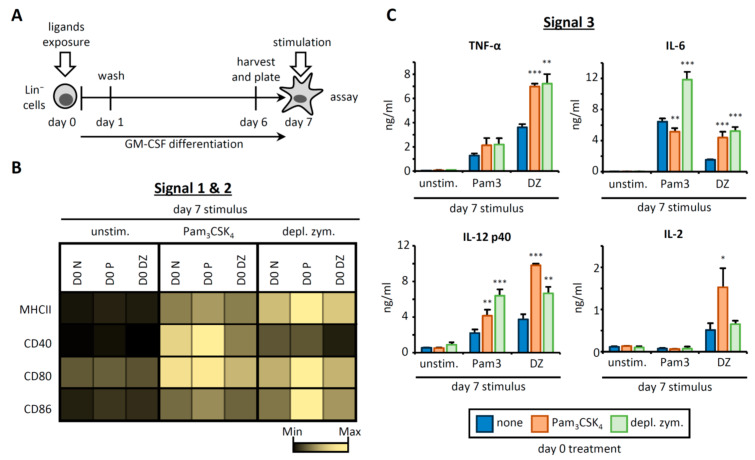
Pam_3_CSK_4_- or depleted zymosan-stimulated hematopoietic stem and progenitor cells (HSPCs) generate antigen presenting cells (APCs) with an altered phenotype in signals 1, 2, and 3. (**A**) Schematic protocol of in vitro HSPC differentiation. Purified Lin^−^ cells from bone marrow of C57BL/6 mice were treated with Pam_3_CSK_4_, depleted zymosan, or nothing (none) during the first 24 h of culture, washed thoroughly to remove any remaining stimuli and then continued in culture with GM-CSF for a further 6 days to derive APCs. At day 6, adherent cells were recovered from the cultures, counted and plated at equal numbers for 24 h, and then stimulated with Pam_3_CSK_4_, depleted zymosan, or nothing (unstimulated) for 24 h to assess: (**B**) the expression of MHCII (signal 1) and costimulatory molecules (CD40, CD80, and CD86; signal 2) on CD11b^+^ CD11c^+^ APCs by flow cytometry (D0, day 0; N, none; P, Pam_3_CSK_4_; DZ, depleted zymosan), and (**C**) cytokine production (TNF-α, IL-6, IL-12 p40, and IL-2; signal 3) in the supernatants from the APC cultures by ELISA. Colormap is based on min-max values per row using the Mean Fluorescence Intensity (MFI) values obtained with the specific antibodies and the isotype controls (shown in Appendix A Figure A1C). Cytokine data represent means ± SD of triplicate cultures, * *p* < 0.05, ** *p* < 0.01, *** *p* < 0.001 with respect to day 0 none. Results shown are from one experiment representing three–five independent experiments.

**Figure A1:**
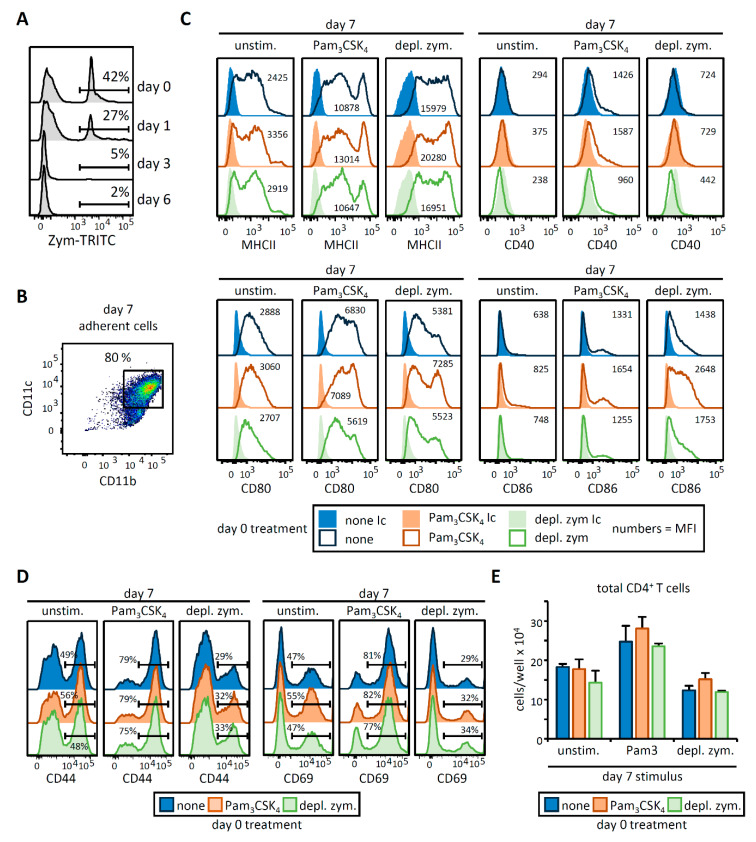
(**A**) Percentage of fluorescently (TRITC) labeled zymosan particles present in the differentiation cultures shown in Figure 1 at different time points measured by flow cytometry. (**B**) CD11b and CD11c expression by the adherent cells recovered from the cultures at day 7 (**C**) Histograms and MFIs (numbers) corresponding to data shown in Figure 1B colormap. (**D**) Histograms and percentage of CD4^+^ T cells expressing CD44 or CD69 from cocultures obtained as indicated in Figure 2A. (**E**) Total CD4^+^ T cell numbers in the cocultures obtained as in Figure 2B, data represent means ± SD of triplicate cultures. Results shown are from one experiment representing three independent experiments.

To investigate the functional consequences of HSPC exposure to a TLR2 or Dectin-1 agonist on the APCs they produce, we designed the experimental approach described in Figure 1A. Briefly, HSPCs (Lin^−^ cells) were purified from mouse bone marrow and cultured with GM-CSF to induce differentiation to APCs. Lin^−^ cells were treated with Pam_3_CSK_4_ or depleted zymosan (a Dectin-1-activating β-glucan particle), or untreated (none) during the first 24 h of culture (day 0), and then washed thoroughly to remove any remaining microbial components prior to continued culture with GM-CSF to derive APCs (Figure 1A). We verified that β-glucan particles were mostly absent in the cultures at day 3 by using fluorescently labeled zymosan (Appendix A Figure A1A). On day 6, adherent cells were recovered from the cultures, counted, plated overnight, and stimulated (day 7) with Pam_3_CSK_4_ or depleted zymosan, or unstimulated. We then assessed their expression of surface molecules and cytokines involved in antigen presentation and T cell activation.

Expression of MHCII (required for signal 1) and costimulatory molecules (CD40, CD80, and CD86; signal 2) on CD11b^+^ CD11c^+^ cells was assessed by flow cytometry (Figure 1B, Appendix A Figure A1B,C). As expected, day 7 stimulation of APCs with Pam_3_CSK_4_ or depleted zymosan upregulated the expression of most of these surface molecules in comparison to unstimulated APCs. Interestingly, day 0 treatment of HSPCs with Pam_3_CSK_4_ increased the expression of MHCII, CD80, and CD86 on day 7-stimulated APCs, while depleted zymosan treatment of HSPCs on day 0 induced more subtle changes in day 7-stimulated APCs. Next, production of cytokines (signal 3) was assessed in the culture supernatants of day 7 APCs (Figure 1C). Day 0 treatment of HSPCs with Pam_3_CSK_4_ or depleted zymosan increased the production of TNF-α, IL-6 and IL-12 p40 (the common subunit for IL-12 and IL-23) by day 7-stimulated APCs, with the exception of Pam_3_CSK_4_-stimulated APCs derived from day 7 Pam_3_CSK_4_-exposed HSPCs, which produced significantly less IL-6. As previously described, IL-2 was only secreted in response to depleted zymosan [11], and surprisingly, only the treatment of HSPCs with Pam_3_CSK_4_ boosted its production. Overall, these data illustrate that HSPC treatment with Pam_3_CSK_4_ or depleted zymosan modifies the T cell-activating signals 1, 2, and 3 of the APCs derived from them.

### 3.2. OVA-Loaded APCs Derived from HSPCs Exposed to Pam_3_CSK_4_ or Depleted Zymosan Prime Altered OVA-Specific CD4 T Cell Responses

**Figure 2:**
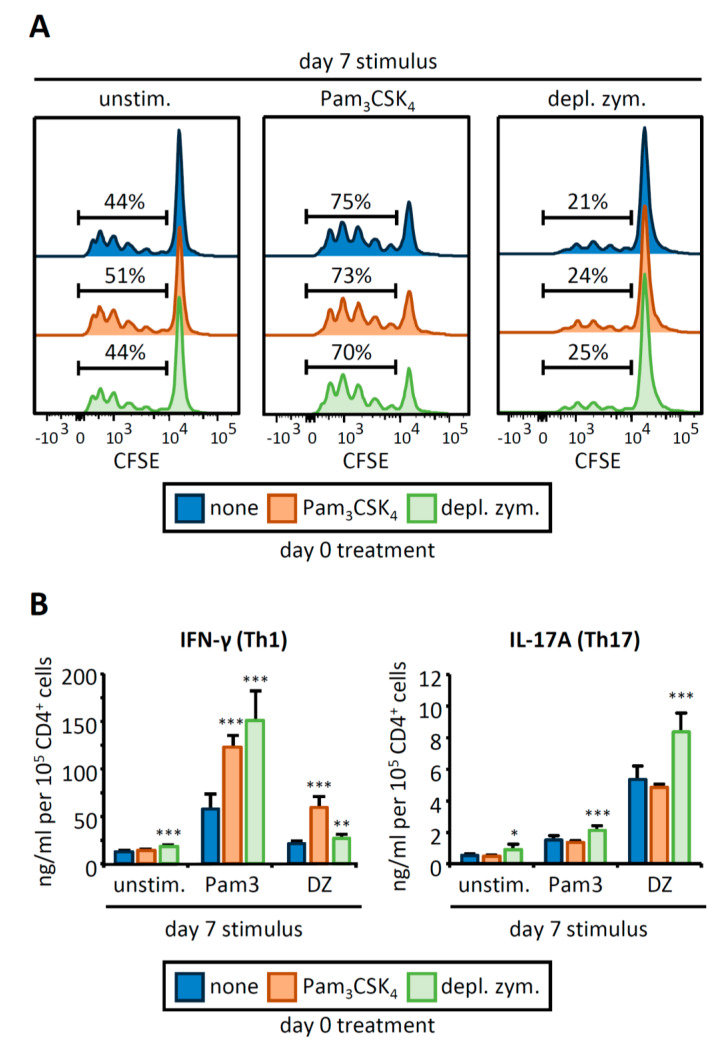
APCs derived from HSPCs stimulated with Pam_3_CSK_4_ or depleted zymosan prime enhanced Th1 and Th17 responses in OVA-specific CD4 T cell cocultures. APCs obtained as shown in Figure 1 were loaded with the OVA_323–339_ peptide and cocultured with CFSE-labeled CD4 T cells isolated from OT-II mice at 1:5 ratio (APC:T cell). Following three days of coculture, T cells were harvested for (**A**) flow cytometry analysis of proliferating CD4^+^ T cells and (**B**) cytokine production (IL-17A and IFN-γ) after 24 h of restimulation with PMA and ionomycin. Cytokine data was normalized by CD4 T cell numbers (shown in Figure A1E) and represent means ± SD of triplicate cultures, * *p* < 0.05, ** *p* < 0.01, *** *p* < 0.001 with respect to day 0 none. Results shown are from one experiment representing three independent experiments.

We next examined whether the changes observed in the APC phenotype could impact the proliferation and activation of CD4 T cells. To address this, day 7 APCs were loaded with an OVA peptide and cocultured with CFSE-labeled OVA-specific CD4 T cells isolated from OT-II transgenic mice (Figure 2). T cells primed by day 0 control APCs stimulated with Pam_3_CSK_4_ (day 7) were more proliferative and expressed higher levels of the activation markers CD44 and CD69 than T cells primed by unstimulated APCs (Figure 2A, Appendix A Figure A1D). Conversely, day 7 stimulation of APCs with depleted zymosan decreased T cell proliferation and downregulated CD44 and CD69.

Day 0 Pam_3_CSK_4_ or depleted zymosan treatment of HSPCs did not cause significant changes in T cell proliferation, T cell numbers, or activation marker expression (Figure 2A and Appendix A Figure A1D,E). To evaluate Th1 and Th17 responses, we measured IFN-γ and IL-17A production by the CD4 T cells (Figure 2B). Consistent with previous reports [12,13], Pam_3_CSK_4_-stimulated APCs induced IFN-γ production by CD4 T cells, whereas depleted zymosan-stimulated APCs induced IL-17A production by CD4 T cells. APCs derived from HSPCs treated with both agonists more potently stimulated IFN-γ production by CD4 T cells in all the conditions studied, whereas only APCs derived from depleted zymosan-treated HSPCs significantly increased IL-17A production by CD4 T cells (Figure 2B). These results indicate that HSPCs programed by Pam_3_CSK_4_ or depleted zymosan can produce trained APCs capable of more strongly priming Th1 and Th17 responses in CD4 T cell cocultures.

### 3.3. HSPC Exposure to Pam_3_CSK_4_ or Depleted Zymosan Alters MHCII Levels, Co-Stimulatory Molecule Expression, and Cytokine Release by APCs Derived from Them In Vitro after C. albicans Yeast Stimulation

**Figure 3:**
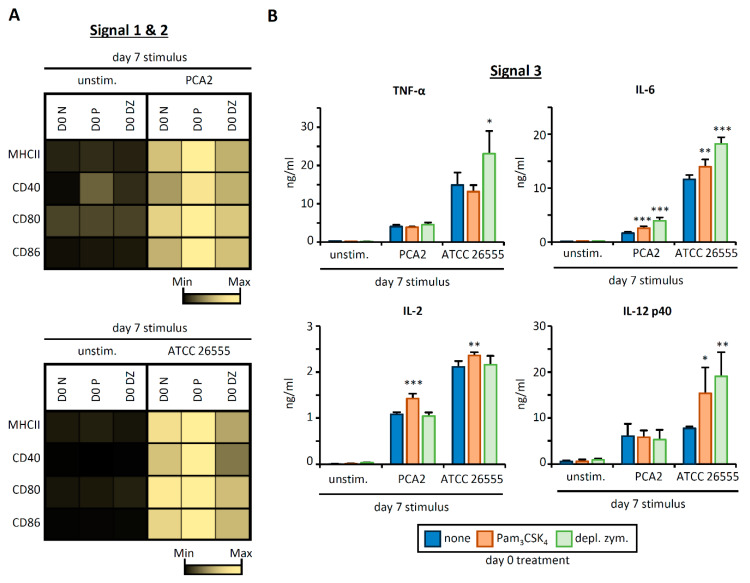
Pam_3_CSK_4_- or depleted zymosan-stimulated HSPCs generate APCs with an altered phenotype in Signals 1, 2 and 3 after *C. albicans* stimulation. Purified Lin^−^ cells from bone marrow of C57BL/6 mice were treated with Pam_3_CSK_4_, depleted zymosan, or nothing (none) during the first 24 h of culture, washed thoroughly to remove any remaining stimuli, and then continued in culture with GM-CSF for a further six days to derive APCs. At day 6, adherent cells were recovered from the cultures, counted and plated at equal numbers for 24 h, and then stimulated with inactivated yeasts of *C. albicans* from the non-virulent strain PCA2 or the virulent strain ATCC 26555, or nothing (unstimulated) for 24 h to assess: (**A**) the expression of MHCII (signal 1) and costimulatory molecules (signal 2) on CD11b^+^ CD11c^+^ APCs by flow cytometry (D0, day 0; N, none; P, Pam_3_CSK_4_; DZ, depleted zymosan) and (**B**) the cytokine production (signal 3) in the supernatants from the APC cultures by ELISA. Colormap is based on min-max values per row using the MFI values obtained with the specific antibodies and the isotype controls (shown in Appendix A Figure A2A). Cytokine data represent means ± SD of triplicate cultures, * *p* < 0.05, ** *p* < 0.01, *** *p* < 0.001 with respect to day 0. Results shown are from one experiment representing three independent experiments.

**Figure A2:**
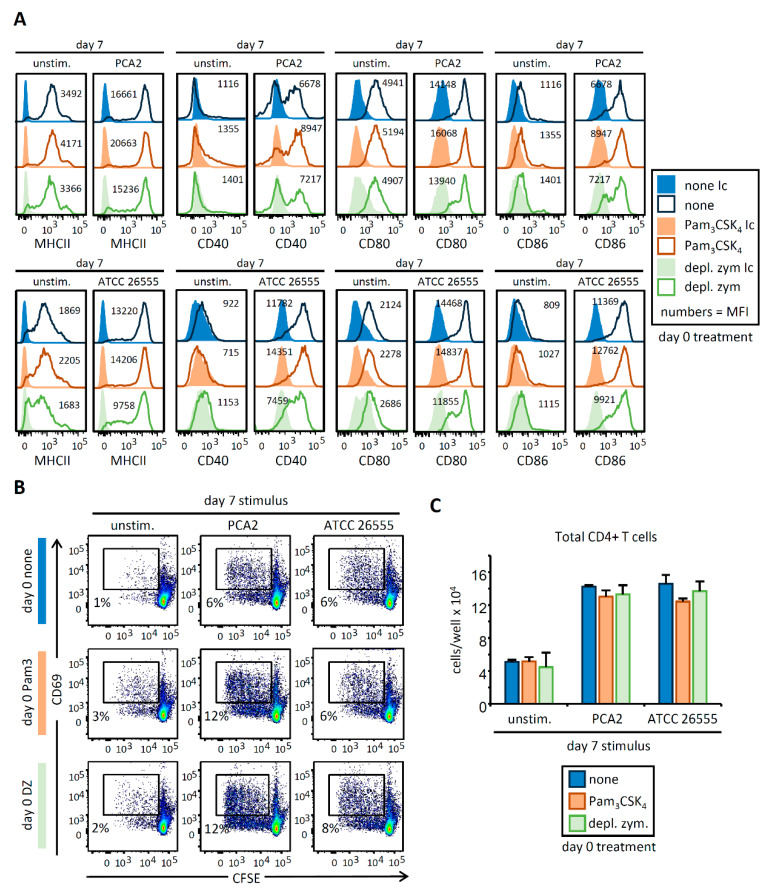
(**A**) Histograms and MFIs (numbers) corresponding to data shown in Figure 3A colormaps. (**B**) Percentage of proliferating CD4^+^ T cells expressing CD69 from cocultures obtained as indicated in Figure 4A. (**C**) Total CD4^+^ T cell numbers in the cocultures obtained as in Figure 4B, data represent means ± SD of triplicate cultures. Results shown are from one experiment representing three independent experiments.

In order to investigate the functional responses of APCs derived from Pam_3_CSK_4_/depleted zymosan-programed HSPCs to intact microorganisms (which signal through several PRRs simultaneously), we used inactivated yeasts from a non-virulent (PCA2) [14] and a virulent (ATCC 26555) strain of *C. albicans* as day 7 APC stimuli (Figure 3). APC expression of MHCII, CD40, CD80, and CD86 on CD11b^+^ CD11c^+^ cells increased upon stimulation with both strains of *C. albicans*, and consistent with the previous results, day 0 treatment of HSPCs with Pam_3_CSK_4_ further increased the expression of these proteins. In contrast, depleted zymosan treatment of HSPCs at day 0 did not change the induction of these APC surface molecules by the non-virulent strain, but decreased their induction by the virulent strain (Figure 3A, Appendix A Figure A2A).

Day 0 treatment of HSPCs with Pam_3_CSK_4_ increased the production of IL-6 and IL-2 by APCs stimulated with both the non-virulent and virulent strains, and increased IL-12 p40 production by APCs stimulated with the virulent strain, but did not have any effect on TNF-α production by either strain (Figure 3B). On the other hand, day 0 treatment of HSPCs with depleted zymosan potently augmented the production of IL-6 by APCs stimulated with both the non-virulent and virulent strains, and TNF-α and IL-12 p40 in response to the virulent strain, but did not impact IL-2 production (Figure 3B).

These results further demonstrate that HSPC programing by Pam_3_CSK_4_ or depleted zymosan impacts the T cell-activating signals 1, 2, and 3 of the APCs derived from them, and that changes in cytokine responses are dependent on the pathogen strain the APCs are responding to.

### 3.4. C. albicans Stimulation of APCs Derived from HSPCs Exposed to Pam_3_CSK_4_ or Depleted Zymosan Enhances Th1 and Th17 Responses in a Strain-Dependent Manner

**Figure 4:**
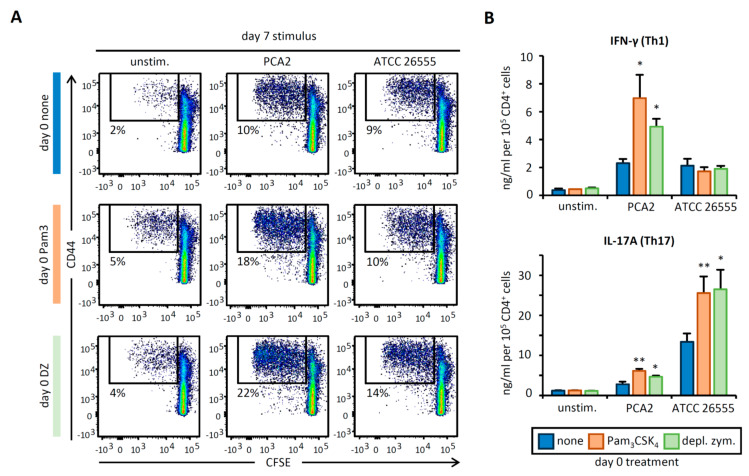
*C. albicans*-stimulated APCs derived from Pam_3_CSK_4_- or depleted zymosan-exposed HSPCs prime enhanced Th1 and Th17 responses in CD4 T cell cocultures. APCs obtained as in Figure 3 were cocultured with CFSE-labeled CD4 T cells isolated from naïve C57BL/6 mice at 1:5 ratio (APC:T cell). Following 4 days of coculture, T cells were harvested for (**A**) flow cytometry analysis of proliferating CD4^+^ T cells expressing CD44 and (**B**) cytokine production (IL-17A and IFN-γ) after 24 h of restimulation with PMA and ionomycin. Cytokine data were normalized by CD4 T cell numbers (shown in Figure A2C) and represent means ± SD of triplicate cultures, * *p* < 0.05, ** *p* < 0.01 with respect to day 0. Results shown are from one experiment representing three independent experiments.

We next evaluated how *C. albicans* stimulation of APCs derived from programed HSPCs modifies the proliferation and activation of CD4 T cells isolated from naïve wild-type mice. Under these conditions, *C. albicans* cells act as multi-antigen sources as well as activators of APC PRRs. Day 7 stimulation of APCs with both strains of *C. albicans* increased total T cell numbers and the percentage of proliferating CD44^+^ and CD69^+^ T CD4 cells in comparison to unstimulated APCs. Day 0 treatment of HSPCs with Pam_3_CSK_4_ and depleted zymosan modestly enhanced T cell proliferation when APCs were stimulated with the non-virulent strain, although T cell numbers in the cultures did not change, and there was little or no effect on T cell proliferation when APCs were stimulated with the virulent strain (Figure 4A, Appendix A Figure A2B,C).

Interestingly, however, IFN-γ production by CD4 T cells was induced by stimulating APCs with both strains of *C. albicans*, and treatment of HSPCs with Pam_3_CSK_4_ and depleted zymosan enhanced IFN-γ production when APCs were stimulated with the non-virulent strain, but not the virulent strain. Moreover, IL-17A production by CD4 T cells, which was greater upon APC stimulation with the virulent strain than the non-virulent strain, was clearly enhanced by treating HSPCs with both Pam_3_CSK_4_ and depleted zymosan at day 0 (Figure 4B). Taken together, these data show that APCs derived from HSPCs programed by Pam_3_CSK_4_ or depleted zymosan have an improved ability to induce Th1, Th17, or both responses, depending on the pathogen strain to which the APCs are responding.

## 4. Discussion

Innate immune memory has raised significant interest in the scientific community since it was discovered that the BCG vaccine induces a primed state in monocytes. This effect, often called training, can also be triggered by fungal pathogens, such as *C. albicans*; upon recognition of fungal β-glucans, Dectin-1 signaling induces metabolic and epigenetic changes that confer protection against the same or unrelated pathogens [15]. 

Mechanistically, the protection conferred by myeloid training may be due to: (i) enhanced nonspecific innate immune responses of monocytes or cells derived from them, e.g., increased cytokine production, which could boost the recruitment and activation of other immune cells, contributing to pathogen clearance [15]; (ii) HSPC programing, which increases myelopoiesis and improves the functional phenotype of the macrophages produced [5,7,8,16], explaining the long-lasting effects of trained immunity; and (iii) modification of the T cell priming potential of antigen presenting cells, which could enhance adaptive T cell responses, thus bridging innate training with an improved adaptive response. The latter idea has not previously been formally demonstrated, although some previous studies have supported this idea, including the observation that bone marrow-derived APCs from mice exposed to the filarial nematode glycoprotein ES-62 prime anti-inflammatory responses, and that PGE_2_ released by UV irradiation induces dendritic cell migration defects due to epigenetic changes in HSPCs [17,18,19].

We therefore decided to study whether stimulation of HSPCs with Dectin-1 or TLR2 ligands could alter the functional phenotype of the APCs derived from them and subsequently have an impact in T cell activation. We used GM-CSF to derive APCs from HSPCs, as it is the most widely used growth factor to study the biology of these cells [20]. While M-CSF drives macrophage differentiation, GM-CSF induces the development of monocyte-derived cells with a much higher antigen presentation capacity. More importantly, GM-CSF has been used for the development of dendritic cell vaccines, which are prepared by culturing peripheral blood from patients in vitro for autologous transplantation, although there is still some debate about naming these APCs “dendritic cells” [20,21]. GM-CSF cultures are heterogeneous [20,22]; however, by starting from HSPCs, instead of total bone marrow, and in our culture conditions, about 80% of the cells are CD11b^+^ CD11c^+^, which, depending on their activation state, can show differences in the expression of functional molecules. Here, we showed that induction of MHCII (required for antigen presentation; signal 1) and costimulatory molecules (CD40, CD80 and CD86; signal 2) on APCs is mostly enhanced by TLR2 stimulation of HSPCs, while Dectin-1 stimulation decreases or does not change them. We also observed changes in the levels and types of cytokines produced by APCs (signal 3), which are dependent on the combination of the stimuli used for HSPC programing and for APC stimulation. Interestingly, while TLR2-stimulated HSPCs give rise to tolerized macrophages in M-CSF cultures [4], here, we showed that GM-CSF-induced differentiation of TLR2-stimulated HSPCs gives rise to trained APCs (enhanced response). Antigen uptake and presentation could also be influenced by HSPC programing. Although previous studies showed minor changes in the phagocytic ability of macrophages derived from programed HSPCs [4,5], we cannot rule out differences in *C. albicans* cell uptake by APCs. However, here, we also employed OVA peptide as an antigen. Since it is directly loaded on to the MHCII molecules, T cell responses would not be affected by differences in antigen uptake and presentation by APCs. Our results show that different Th1 and Th17 outcomes may be achieved by varying the set of PRR ligands used for HSPC programing and for APC stimulation, although future experiments will be needed to better define the factors contributing to Th1 and Th17 polarization. Interestingly, depending on the strain of *C. albicans* used to stimulate APCs, a different Th response was observed. Independent of the stimulus used to treat HSPCs, IFN-γ production was only enhanced when APCs derived from programed HSPCs were stimulated with a nonvirulent strain, while more potent IL-17 production was achieved after stimulating APCs derived from programed HSPCs with a virulent strain of *C. albicans*. Further experiments will evaluate whether HSPC programing could also influence CD8 T cell responses and whether innate immune memory impacts the generation of long-lasting memory T cells.

## 5. Conclusions

Our results highlight the importance of the modifications caused by microbial signals detected by HSPCs, which can be sustained during their differentiation towards APCs and subsequently enhance T cell responses. Understanding the mechanisms underlying innate immune memory in HSPCs will provide us with new strategies for the development of better tailored anti-microbial vaccines.
